# Nanomechanical variability in the early evolution of vertebrate dentition

**DOI:** 10.1038/s41598-022-14157-2

**Published:** 2022-06-17

**Authors:** Mohammad Shohel, Kamal K. Ray, Alexei V. Tivanski, Neo E. B. McAdams, Alyssa M. Bancroft, Bradley D. Cramer, Tori Z. Forbes

**Affiliations:** 1 Department of Chemistry, University of Iowa, Iowa City, IA 52242 USA; 2 Department of Geosciences, Texas Tech University, Lubbock, TX 79409 USA; 3 Iowa Geological Survey, University of Iowa, Iowa City, IA 52242 USA; 4 Department of Earth and Environmental Sciences, University of Iowa, Iowa City, IA 52242 USA

**Keywords:** Geochemistry, Palaeontology, Materials chemistry

## Abstract

Conodonts are an extinct group of primitive jawless vertebrates whose elements represent the earliest examples of a mineralized feeding apparatus in vertebrates. Their relative relationship within vertebrates remains unresolved. As teeth, conodont elements are not homologous with the dentition of vertebrates, but they exhibit similarities in mineralization, growth patterns, and function. They clearly represent an early evolutionary experiment in mineralized dentition and offer insight into analogous dentition in other groups. Unfortunately, analysis of functional performance has been limited to a handful of derived morphologies and material properties that may inform ecology and functional analysis are virtually unknown. Here we applied a nanoscale approach to evaluate material properties of conodont bioapatite by utilizing Atomic Force Microscopy (AFM) nanoindentation to determine Young’s modulus (E) along multiple elements representing different ontogenetic stages of development in the coniform-bearing apparatus of *Dapsilodus obliquicostatus*. We observed extreme and systematic variation in E along the length (oral to aboral) of each element that largely mirrors the spatial and ontogenetic variability in the crystalline structure of these specimens. Extreme spatial variability of E likely contributed to breakage of elements that were regularly repaired/regrown in conodonts but later vertebrate dentition strategies that lacked the ability to repair/regrow likely required the development of different material properties to avoid structural failure.

## Introduction

The origin of dentition is a central question in the evolution of vertebrates^1,2^ and conodonts provide an important window into the early evolutionary development of mineralized feeding mechanisms^1,3–5^. The mineralized components of conodonts consisted of morphologically diverse tooth-like elements composed originally of hydroxyapatite that formed an oropharyngeal raptorial array within the animal that was used to capture and process prey items^6,7^. The morphology of the early conodont apparatus was limited to simple cone-like (coniform) elements and later euconodonts developed increasingly complex assortments of morphologically diverse elements within a single apparatus that rivalled the diversity of dentition in even the most complex crown-group vertebrates^3,8^. Nearly all investigations of the functional performance of individual conodont elements have focused on later derived groups with a morphologically complex feeding apparatus and primarily utilized wear patterns to investigate function during occlusion^1,9^. Species that contain multiple coniform morphologies, but without more complex and derived platform or ramiform elements, were a significant component of total conodont diversity from the late Cambrian into the Early Devonian^10^, yet even less is known about the functional performance and utilization of these evolutionarily more primitive forms. Tomographic studies and 2-D finite element modelling of some coniform elements have begun to investigate functional performance in these plesiomorphic morphologies^11,12^, and further finite element modelling has been conducted on more derived morphologies as well^13–15^. However, these studies have been limited by the lack of known values for material properties of conodont bioapatite and several critical values such as Young’s modulus have had to be estimated from measurements of extant vertebrates^11–15^.

**Figure 1:**
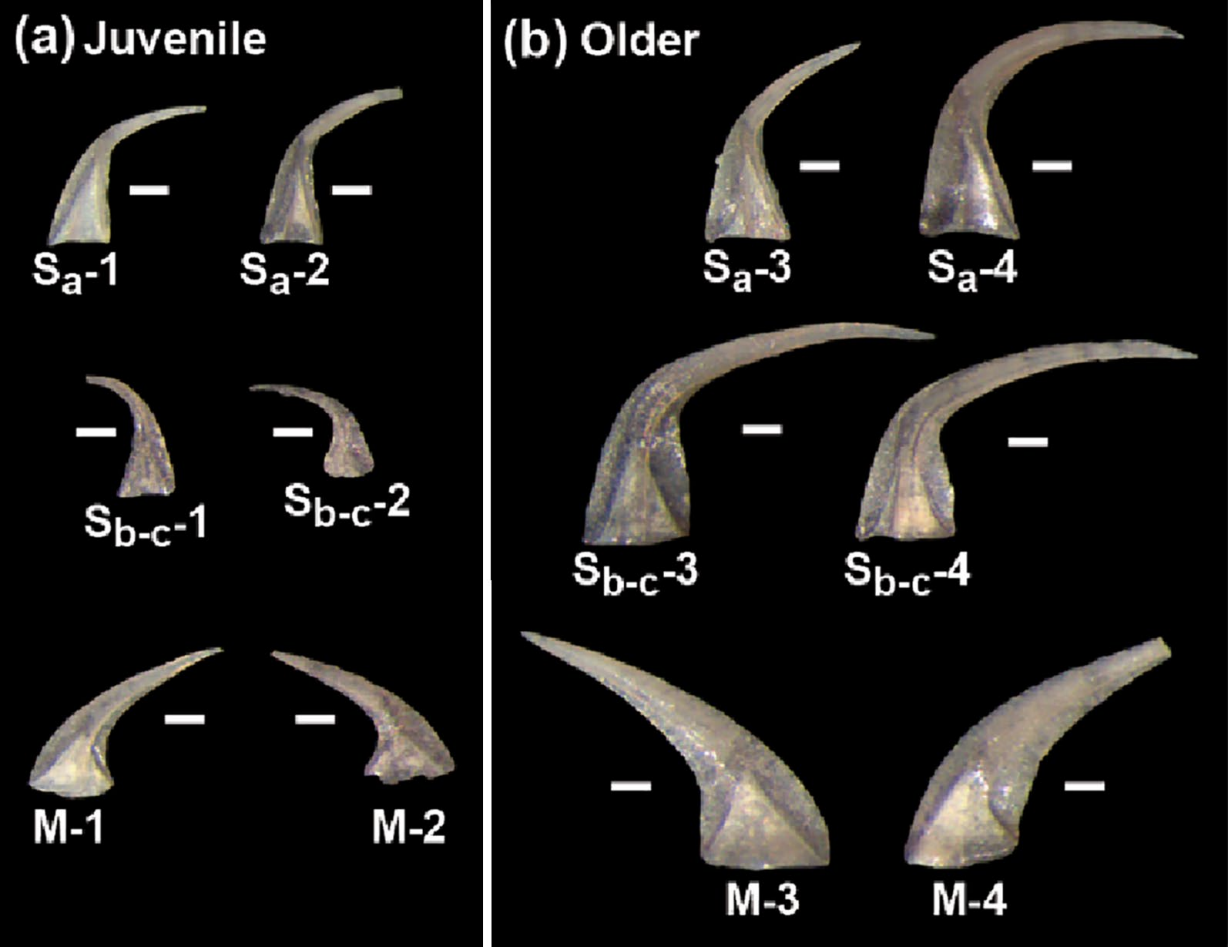
Reflected light photomicrographs of specimens of Dapsilodus obliquicostatus sampled in this study including all element types (S_a_, S_b-c_, and M elements). All scale bars are 100 μm and all images are scaled to the same size. These are the same specimens previously characterized by micro-X-ray diffraction (μXRD)^21^.

Young’s modulus (E), or the elastic modulus, is a measure of a material’s resistance to deformation under load and is more generally referred to as a material’s stiffness. Stiff materials have a higher E (e.g., diamond = 1064–1217 GPa), while soft materials that exhibit larger elastic deformation have a lower value (e.g., rubber = 0.01–0.1 GPa). The stiffness of the materials that form any feeding apparatus will necessarily provide a first-order control on functional performance and Young’s modulus is a critical value^16^ that has previously been unknown for conodont bioapatite. Pure single crystalline apatite is typically stiffer (hydroxyapatite = 62.0–150.4 GPa) than bioapatite, which is a nanocomposite of apatite crystallites in an organic matrix, and measured values of E from vertebrate bioapatite range from as low as 2.40 GPa in bones up to 120 GPa in teeth^16–20^. Limited information is available regarding the spatial variability of Young’s modulus across the functional surface in vertebrate teeth. The few studies conducted demonstrate that there are clearly variations in E across the functional surface (buccal to lingual) and along the apical-cervical axis in bovine dentine^19^ and human molars^20^. Similar stiffness mapping of enamel and dentine in dinosaur and crocodylomorph teeth shows considerable variability of appoximately 25% of the maximum values of E for each material^18^. The range of E appears to be smaller (25%-30%) across the functional surface of vertebrate teeth compared to longitudinal variations from the functional surface to the enamel-dentine junction, which can exceed 50%^18,20^. Conodont bioapatite is composed of distinct tissue types, each composed of varying amounts and arrangements of hydroxyapatite nanocrystallites in an organic matrix^8^. The majority of each conodont element is composed of crown tissue with basal tissue limited to the region that was presumably permanently attached to the surrounding soft tissue^4,12,13^. Crown tissue is composed of hyaline lamellar crown tissue and white matter (albid), each of which have their own microstructure and crystallographic properties^6,8,21^. Conodont elements grow through the apposition of lamellae with nanocrystallites typically arranged perpendicular or parallel to the growth lamellae, although a variety of growth patterns have been observed^8^. As the first investigation of Young’s modulus in conodont bioapatite we used Atomic Force Microscopy (AFM) nanoindentation to evaluate the mechanical property of lamellar crown tissue in 12 specimens of *Dapsilodus obliquicostatus* (Fig. 1) including multiple element types in the apparatus (S_a_, S_b-c_, and M) and from different ontogenetic stages (juvenile vs. older). All of the specimens (Fig. 1) studied here were previously characterized utilizing micro-X-ray diffraction (μXRD) techniques^21^, which allows us to directly relate the measured nanomechanical property to crystalline structure of the materials in each element. The new AFM data presented here provide the first documentation of this important mechanical property (E) of conodonts and demonstrate a close relationship between variations in Young’s modulus, ontogenetic development, and crystallographic structure of conodont bioapatite.

## Results

Stiffness along the length of each studied element was quantified by calculating Young’s modulus values (E) at five locations along the element by fitting indentation force plots to a Johnson-Kendall-Roberts (JKR) model (see “Methods” and SI for more details)^22–25^. The substrate-induced effects on the measured values were negligible under our experimental conditions because the typical maximum thickness of a fossil conodont (ca 10–100 μm) is many orders of magnitude larger than a typical indentation depth (ranging from 0.001 to 0.004 µm). Surface morphology was characterized using the AFM amplitude images and nano-scale roughness and variation in surface topology were observed for all samples (SI). However, no systematic variations were observed along the length of elements. Roughness and other topological features observed on the specimen surfaces are due to wear, neo-crystal growth, or post-mortem diagenesis^26,27^.

**Figure 2:**
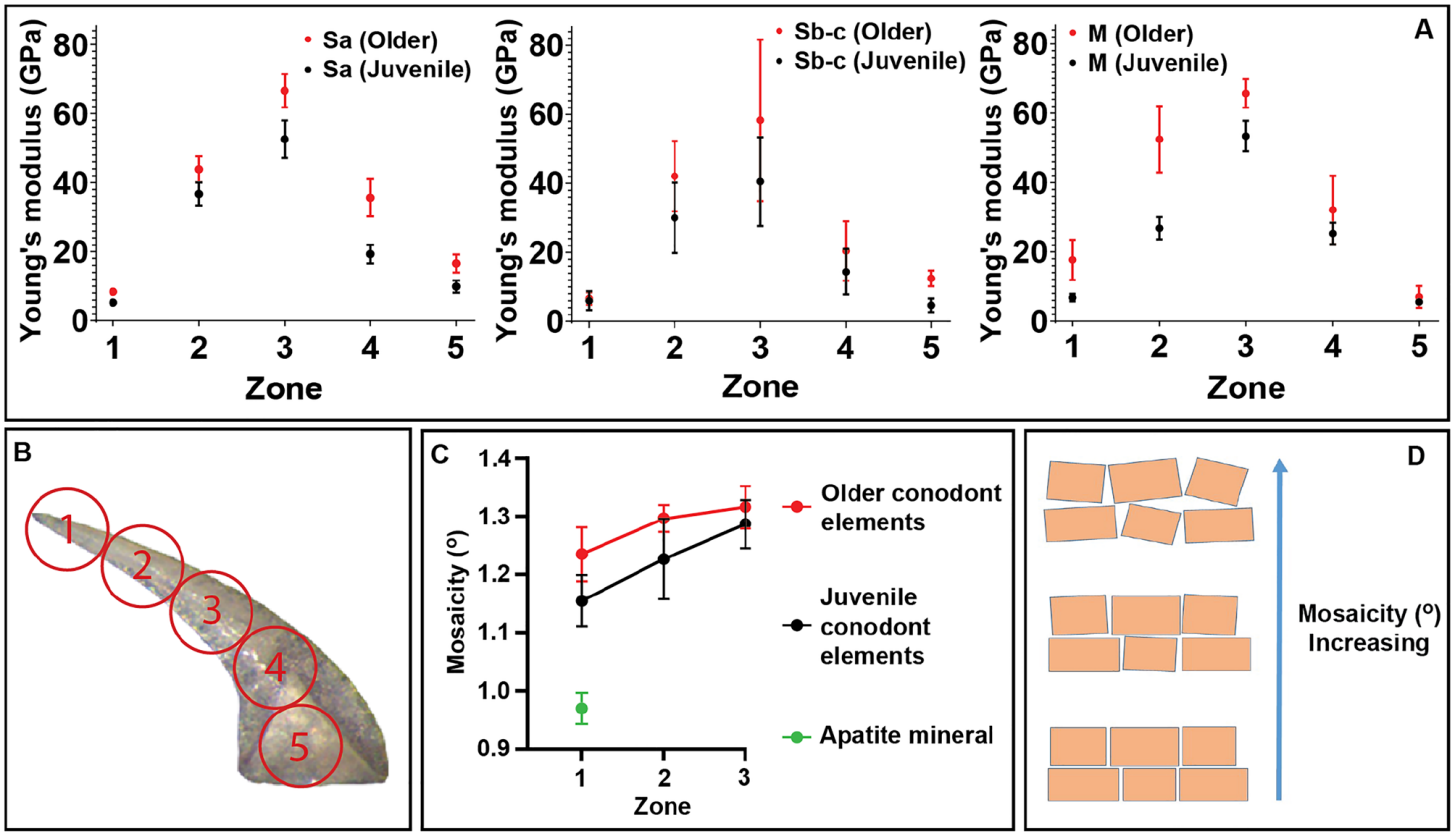
(**A**) Young's modulus (average and one standard deviation) by element type and position along each element. (**B**) Measurements taken along the length (oral to aboral, 1 through 5, respectively). (**C**) Mosaicity along the length of elements in zones 1–3. Mosaicity can only be calculated in those areas exhibiting single crystal diffraction patterns^21^ and is a measure of nanocrystallite disordering (**D**) where more orderly arrangements have a lower mosaicity value. Comparing (**A**) and (**C**) there is a clear correlation between increasing mosaicity and increasing Young’s modulus as well as consistency between juvenile and older specimens.

We observed systematic variation in E along the oral-aboral axis of each element sampled, where values progressively increase from the tip to roughly the midpoint and then decrease from the midpoint to the base (Fig. 2). Values of E for the lamellar crown tissue exhibit an unexpected degree of variability and range by greater than an order of magnitude from 4 to 83 GPa. Older specimens are also systematically more stiff (higher E) than their juvenile counterparts across the length of all element types (S_a_, S_b-c_, M). These variations in material stiffness largely mirror the crystallographic variability in these specimens^21^ where the degree of disordering in the crystallites (mosaicity) is directly correlated with the material stiffness in all available data except for the base of the elements where mosaicity cannot be calculated^21^ (Fig. 2).

## Discussion

The relationships between crystallographic arrangement and material stiffness in Fig. 2 are both unexpected and difficult to explain. In most vertebrate bioapatites, the ratio of total amount of apatite crystals to organic matrix (or level of mineralization) is typically a first-order control on the stiffness of the material such that materials with higher mineral density (i.e. crystals/unit volume) are typically stiffer^16,19^. The degree of mosaicity should therefore directly impact mineral density such that areas where crystallites are better ordered (i.e. lower mosaicity) should have higher mineral density and therefore higher E, but we see the opposite in the lamellar crown tissue where lower mosaicity at the tips corresponds to lower values of E (Fig. 2). This is also true in the overall comparisons between younger and older specimens as well where mosaicity (disorder) increases with age along with E.

Young’s modulus (E) is primarily a measure of the interatomic bond forces of a material^28–30^, and the classical Hall–Petch (H–P) relationship suggests that the yield strength of a polycrystalline material should increase with decreasing grain size. However, in nanocrystalline materials where individual grains are in the order of tens of nm or less, an inverse H–P relationship develops where decreasing grain size reduces the yield strength of the material^29–31^. Conodont nanocrystallite sizes vary from a few nm in the short dimension to nearly 100 nm in the long dimension^32–34^, and are within the range of size where changes in the H-P relationship begin to become apparent. This inverse H–P relationship is the result of relative increases in the volume of grain boundaries and triple junctions in nanocrystalline materials, where the interactions between grains become more significant than the properties of individual grains themselves. However, the precise mechanism responsible for the inverse H–P relationship in nanomaterials remains elusive, and it is unclear if this phenomenon is the result of changes in dislocation motion, diffusion processes, grain-boundary shearing, or composite material behavior^31^.

This inverse H–P relationship, where decreasing grain size will reduce E, is one possible explanation for the data presented in Fig. 2. If there was an increase in overall grain size from the oral tip to the central curve (midpoint) of each specimen, that would have the impact of increasing E along the same path. Similarly, if there were a general increase in grain size with age (ontogeny) of the specimen, that would also have the same impact and increase E with age. Therefore, without further testing to explicitly evaluate nanocrystaline grain size in these speciments, the impact of grain size, via the inverse H-P relationship, remains a possible explanation for the variability in E measured in our samples.

Another possible cause of the highly variable E, and one supported by the data in Fig. 2, is the relationship between crystallographic alignment (mosaicity) and the ability to translate force along grain boundaries. Low mosaicity (low disordering) of nanocrystallites will tend to increase alignment of preferential planes of weakness, where grain boundaries are better aligned over larger distances, and therefore allow for force to be translated more effectively throughout the material^29,31,35^. There is a close correlation between changes in mosaicity and E where lower mosaicity consistently corresponds with lower E, both in position along each element as well as with ontogeny, in all samples measured (Fig. 2). Recent work on a variety of vertebrate enamels has demonstrated that small amounts of crystal ‘misorientation’ confer increased hardness (H) as well as E^35^ and this appears to be supported by the relationship between mosaicity and E seen in our data. Therefore increase in mosaicity (‘misorientation’^35^) from oral tip to the central curve (midpoint), and the increase in mosaicity with age (ontogeny) likely increase E due to the decreased ability to translate force (or propagate dislocations) with increasing disorder (‘mosaicity’) of nanocrystallites. These two effects taken together, of small grain size driving the inverse H–P relationship, and of low mosaicity increasing alignment of grain boundaries, are likely the cause of the highly variable E identified in our samples. Without additional testing we cannot yet determine which of these two effects (grain size or ordering) is playing the largest role in the material strength of these specimens.

The values of E recovered from *D. obliquicostatus* fall within the range of other vertebrate phosphatic feeding elements (teeth) on both the low value and high value ends of the scale (Table 1)^17,18,20,36–38^. Given that lamellar crown tissue of conodonts is analogous to the more derived tooth materials of later vertebrates (enameloid, enamel)^1,3,5,8^, the similarity in the maximum values of this critical material property are striking. However, the extremely low values are surprising and the variation in E over the length of the elements of *D. obliquicostatus* is exceptional. This extreme variability must have had structural consequences. Based upon the distribution of E along the elements in our data, the greatest structural weakness in conodont elements would most likely have been located at the transition from very low E to very high E across the transition from the tip of the element to the point of maximum curvature (Figs. 1 and 2). Importantly, finite element modeling of the shape of coniform morphologies demonstrated that this is also the location of greatest strain^39^ and this combination of structural weakness and strain focusing may help to explain the location of breakage and regrowth in coniform specimens.

**Table 1:** Young's modulus (E) of vertebrate dentition.

Species	Material	E (GPa)	References
*Loxodonta africana *(African elephant)	Dentine	6.6–10.7	^36^
*Monodon monoceros *(Narwhal)	Dentine	8.7–11.9	^36^
*Bas taurus *(European cattle)	Dentine	7.7–14.7	^36,37^
*Homo sapiens *(Human)	Dentine	16.1–23.6	^38^
*Suchomimus tenerensis *(Spinosaurid dinosaur)	Dentine	44–70	^18^
*Sarcosuchus imperator *(Crocodyliform)	Dentine	82–100	^18^
*Sphyrna tiburo *(Bonnethead shark)	Orthodentine	20.8–24.2	^17^
*Carcharias taurus *(Sand tiger shark)	Osteodentine	26.2–30.7	^17^
*Alouatta palliata *(Howler monkey)	Enamel	75–105	^38^
*Carcharias taurus *(Sand tiger shark)	Enameloid	67.9–77.3	^17^
*Sphyrna tiburo *(Bonnethead shark)	Enameloid	67.4–70.4	^17^
*Suchomimus tenerensis *(Spinosaurid dinosaur)	Enamel	69–93	^18^
*Sarcosuchus imperator *(Crocodyliform)	Enamel	92–114	^18^
*Homo sapiens *(Human)	Enamel	80–120^a^	^20^
*Homo sapiens *(Human)	Enamel	47–120^b^	^20^
Dasilodus obliuicostatus (Conodont)	Lamellar crown	4–83	This study

Comparison of Young' modulus (E) of vertebrate dentition from the literature with the results of this study. The orientation of the measured transects in the cited literature varies from across the functional surface in some studies^a^ to variation with depth from the functional surface to the dentine-enamel junction (DEJ)^b^, to “mid-point” values between the functional surface and DEJ.

**Figure 3:**
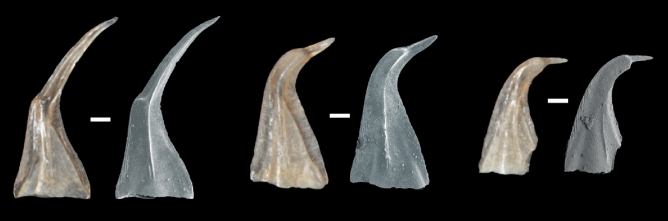
Reflected light photomicrograph and SEM images of broken and regrown S_b–c_ elements of *D. obliquicostatus* from the same stratigraphic interval as sampled in this study. All scale bars at 100 μm and all images are scaled to the same size. The breaks are almost always at the point of maximum curvature and do not appear to be preferentially related to the extent of white matter.

The functional transition in material properties along the length of elements from tip to curve may explain the propensity for coniform elements to break during life at this transition from low to high E. Breakage and regrowth of conodont elements during life is a well-known phenomenon but has rarely been discussed in detail in the literature^40–42^. Breakage in coniform elements almost always occurred near the point of maximum curvature such that the tip is missing but regrowth clearly began for many specimens during the life of the animal. Such breakage and regrowth was apparent in specimens from our collection^43^ (Fig. 3—broken and regrown specimens were not sampled). The relative frequency of specimens recovered that show breakage in-vivo from a given sample is extremely hard to determine based upon breakage alone due to the fact that the breakage we see today may have been taphonomic and significantly post-mortem. Broken and regrown specimens clearly demonstrate that the break occurred during life^42^ and can be used to determine a rough estimate of breakage frequency. However, similar data are almost never reported in the literature. In some of our samples from the Schlamer #1 core used in this study, of the > 300 specimens belonging to *D. obliquicostatus* recovered from a given sample, up to 5% may show breakage and regrowth (Fig. 3).

The ability to regrow/repair parts of an existing element within a mineralized feeding apparatus was a novel feature unique to conodonts that was enabled by their growth strategy of episodic exterior apposition of lamellae most likely while within or covered by the dermis^8,42^. Therefore, the extreme nanomechanical variability in the material properties within single conodont elements that likely promoted their breakage was not catastrophic to the development of conodonts due to their ability to repair these breaks. As helpfully pointed out by Duncan Murdock during the review of this manuscript, this combination of structural weakness and strain focusing near the point of inflection could have been an adaptation to create a ‘break point’ that would avoid catastrophic damage and the loss of the entire element.

The feeding strategies, prey items, and occlusion mechanics of *D. obliquicostatus* remain unknown and it is unclear if the functions of the elements sampled in this study were to grasp, pierce, slice, crush, or crack prey. The function of each element in the feeding apparatus exerted a primary control on the forces the element encountered during feeding and the data presented here provide potential insight into future studies on the structural function of the elements of this species. The variability in E along the length of the elements necessarily impacts the amount of stress each element can withstand. Our data suggest that the oral tip and the point of maximum curvature may have been encountering significantly different stress regimes during feeding, and further finite element and molecular dynamical modelling may provide inferential insight into the ultimate functions of elements in this species.

The mineralized tissues of the conodont feeding apparatus represent an early experiment in the evolution of mineralized dentition. The extreme variability in E within single elements was likely due to the variable patterns of mosaicity and/or the inverse H–P relationship, where increasing disorder (misalignment/mosaicity) and/or increasing nanocrystallite grain size increased E along the length of the element from oral tip to the central curve (midpoint). This nanomechanical variability likely promoted regular breakage, but due to their exterior apposition of lamellae, the conodont animal had the ability to repair such damage to its elements via regeneration. Later vertebrate dentition strategies, particularly those in jaws that could not repair or regrow broken dental elements, likely required different material properties to avoid structural failure.

## Methods

### Specimen details

The details of the specimen, geological setting and extraction procedure can be found in our previous work^21,43^. A total of 181 conodont samples were collected from the Schlamer #1 drillcore, Alexander County, southwestern Illinois, USA. More than 1000 specimens of *D. obliquicostatus* were recovered from the core and all specimens analyzed in this study were recovered from the St. Clair Formation and are well preserved. The samples were thermally unaltered with a conodont colour alteration index (CAI) of 1 indicating a burial temperature no higher than 80 °C^44^. The low CAI of these specimens demonstrates a very low likelihood of pyrolysis of the original organic matter in the bioapatite due to metamorphism. Carbonate and carbonaceous shale samples collected from the drill core were digested using the standard double-buffered formic acid technique^45^. Insoluble residues were further processed by heavy liquid separation utilizing lithium metatungstate (LMT) at a density of 2.83–2.84 kg/L, and the remaining heavy fraction was picked under binocular microscope for conodonts. Twelve specimens of *D. obliquicostatus* were selected to represent a range of ontogenetic development from juvenile to gerontic (Figs. 1 and 3), and ontogenetic development was determined by appearance (size, robustness, apparent wear, transparency).

### Atomic force microscopy experiment

Surface images and mechanical properties were collected using a Molecular Force Probe 3D AFM (Asylum Research, Santa Barbara, CA). Conodont elements depicted in Fig. 1 were adhered to a clean glass surface on a thin layer of epoxy. A tip of a narrow paint brush (Winsor and Newton, size = 0000, series = 111) wetted with n-hexane was used to attach the conodont onto the epoxy surface. The process was conducted under a light microscope to ensure that the sample attached to the surface in the proper orientation. Only the clean surface of the elements was subjected to analysis. The AFM images and nanoindentation measurements were performed at 21 ºC and ambient pressure using a diamond-like-carbon probe (Mikromasch, HQ:XSC11/Hard/Al BS) with a nominal spring constant of 42 N/m and a typical tip radius of curvature of 8 nm. Actual spring constants were determined using the thermal noise method^46^. Before each nanoindentation measurement, the tip calibration was performed on a silicon wafer by determining the deflection sensitivity to convert the force–displacement curve to force versus tip-sample separation plot^47^. Topographic images and nanoindentation studies were performed using intermittent contact mode (AC mode) and contact mode, respectively, at a typical scan rate of 1 Hz. For a typical force plot collection, the AFM probe starts a motion towards the surface from the height of ~ 350 nm above the surface that continues until the predetermined maximum force of 500 nN or 1000 nN is reached. The maximum force of 1000 nN was used as a limit and no obvious mechanical damage was observed on the element surface after a series of repeated force–displacement measurements. AFM imaging after repeated force measurements was used to confirm that the element surface remained intact, with no evidence of plastic deformation, cracks, or cracking marks after applying the maximum loading force. Each conodont element was separated into five different zones for nanoindentation measurements starting from the tip to the basal cavity (oral to aboral direction, Fig. 2B). AFM nanoindentation measurements within the basal cavity were only performed at the top of the cavity. To ensure the reproducibility of the measurements, typically eight to ten repeated force measurements in 10 to 15 different sample locations were collected at each zone. Overall, 400 to 600 individual force plots data were collected at each zone on the sample. The nanoindentations were carried out on relatively plain surfaces avoiding wear, neo-crystal growth, and other unwanted features. In addition, all specimens used in the current study were chosen carefully and exhibited CAI values of 1 to ensure minimal diagenesis.

The additional details about calculating the radius of contact area (a_JKR_), the force arising from interactions between two spheres (F_JKR_), the indentation depth (h_JKR_), the work of adhesion (W) and E are provided in the SI.

## Supplementary Information

Supplementary Information.
