# Supplementary material for: Nanomechanical variability in the early evolution of vertebrate dentition
Source: Sci Rep. 2022 Jun 17;12:10203. doi: 10.1038/s41598-022-14157-2 (PMC9205932; doi:10.1038/s41598-022-14157-2)
Supplement: Supplementary file 1 — Supplementary Information. [file 41598_2022_14157_MOESM1_ESM.docx]

**Supplementary information**

**Nanomechanical Variability in the Early Evolution of Vertebrate Dentition**

Mohammad Shohel^1^, Kamal K. Ray^1^, Alexei V. Tivanski^1^, Neo E.B. McAdams^2^, Alyssa M. Bancroft^3^, Bradley D. Cramer*^4^, Tori Z. Forbes*^1^

^1^Department of Chemistry, University of Iowa, Iowa City, IA-52242

^2^Department of Geosciences, Texas Tech University, Lubbock, TX-79409

^3^Iowa Geological Survey, University of Iowa, Iowa City, IA-52242

^4^Department of Earth and Environmental Sciences, University of Iowa, Iowa City, IA-52242

*Corresponding [authors: tori-forbes@uiowa.edu](mailto:authors: tori-forbes@uiowa.edu); [bradley-cramer@uiowa.edu](mailto:bradley-cramer@uiowa.edu)

**Table of Contents**

1. **Calculation of Young’s Modulus**
2. **AFM Images of Surface**

1. **Calculation of Young’s Modulus**

The equations used to calculate the radius of contact area (a_JKR_), the force arising from interactions between two spheres (F_JKR_), the indentation depth (h_JKR_), the work of adhesion (W) and YM in the JKR models are:

$a_{JKR}=\frac{3RF_{JKR}}{4E^{*}}$ (1)

$h_{JKR}=\frac{a_{JKR}^{2}}{R}-\sqrt{\frac{2\pi Wa_{JKR}}{E^{*}}}$ (2)

$F_{JKR}=F+3\pi WR+ \sqrt{6\pi WR+ {9\pi^{2}W^{2}R}^{2}}$ (3)

$W= -\frac{2}{3}\left( \frac{F_{pull-off}}{\pi R} \right)$ (4)

here R is tip radius of curvature, E^*^ the effective Young’s modulus of the tip and sample determined from the following equation:

$\frac{1}{E^{*}}=\frac{1-\nu_{tip}^{2}}{E_{tip}}+\frac{1-\nu_{crystal}^{2}}{E_{crystal}}$ (5)

where *E*_crystal_ and *E*_tip_ are the Young’s modulus of the sample and tip, respectively. Here, *ν*_tip_ and *ν*_crystal_ are the Poisson’s ratio of the tip and crystal, respectively. The estimated Poisson’s ratio of the tip and crystal are 0.2 and 0.33, respectively.^2-4^ The radius of curvature of the AFM tip and Young’s modulus of tip were 8 nm and 865 GPa, respectively. A representative force versus tip sample separation plot with JKR model fit is shown in Figure S1.


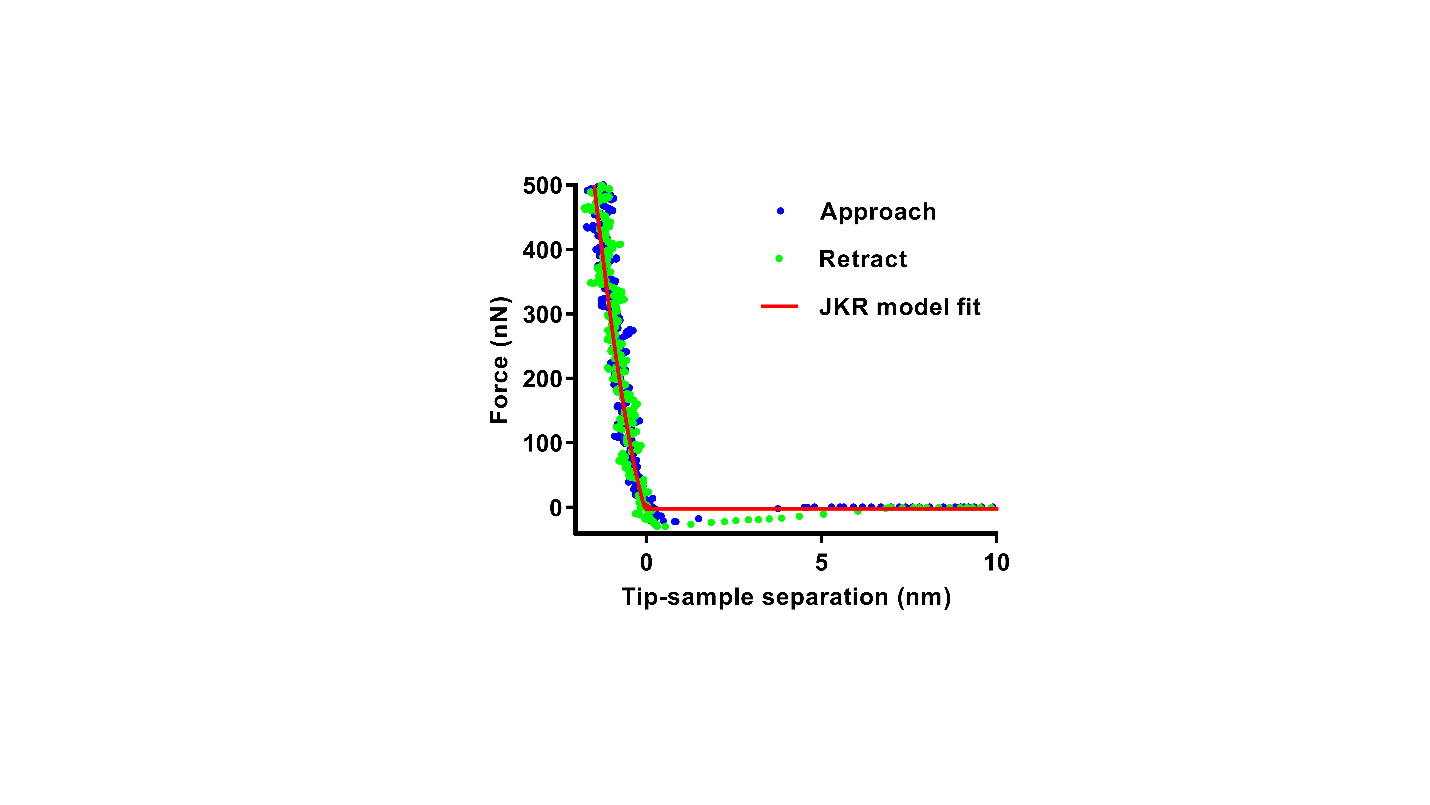


**Figure S1.** A representative force versus tip−sample separation plot is shown at a maximum applied force of 500 nN. The blue and green dots are the approach and retract data, respectively. The solid red line is shown for the JKR fitting of the approach data.

**Table S1.** Young’s modulus values measured using AFM nanoindentation of conodont elements of *Dapsilodus obliquicostatus* at different zones with one standard deviation (average ± SD). All units are in GPa, J and O refers to juvenile and older, respectively.

| Zone | S_a_ (J) | S_a_ (O) | S_b-c_ (J) | S_b-c_ (O) | M (J) | M (O) |
| --- | --- | --- | --- | --- | --- | --- |
| 1 | 5.3±0.6 | 8.4±0.6 | 6.0 ± 2.7 | 6.7 ± 1.8 | 6.8 ± 1.1 | 17.7 ± 5.8 |
| 2 | 36.7±3.3 | 43.9±3.8 | 30.0 ± 10.2 | 41.5±10.4 | 26.8 ± 3.2 | 52.5 ± 9.6 |
| 3 | 52.6±5.4 | 66.6±4.8 | 40.5 ± 12.9 | 65± 13.6 | 53.8 ± 4.4 | 65.7 ± 4.1 |
| 4 | 19.3±2.7 | 35.6±5.4 | 15.4 ± 6.6 | 20.4 ± 8.6 | 25.3 ± 3.1 | 32.1 ± 9.9 |
| 5 | 9.9±1.8 | 16.6±2.6 | 4.7 ± 2.0 | 12.5 ± 2.3 | 5.6 ± 0.6 | 6.9 ± 3.1 |

*J= Juvenile, O= Older

1. **AFM Images of Surface**

**
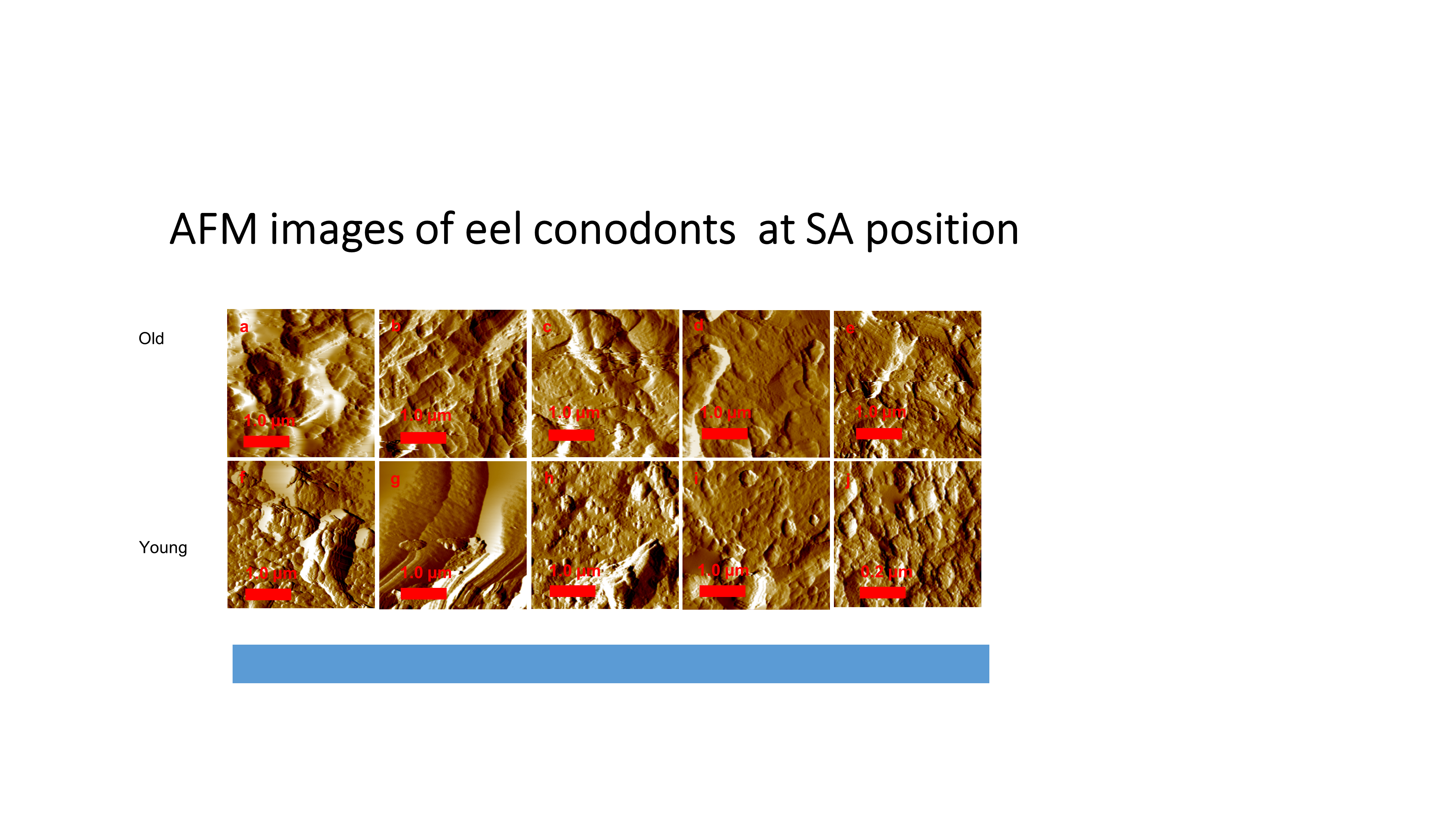
**

**5**

**4**

**3**

**2**

**1**

**O**

**J**

**Figure S2.** Representative AFM amplitude images of S_a_ conodonts, where top (a-e: Zones 1-5) and bottom (f-j: Zones 1-5) rows are for the juvenile and older samples, respectively. The images sequentially show the tip to base segments from left to right.


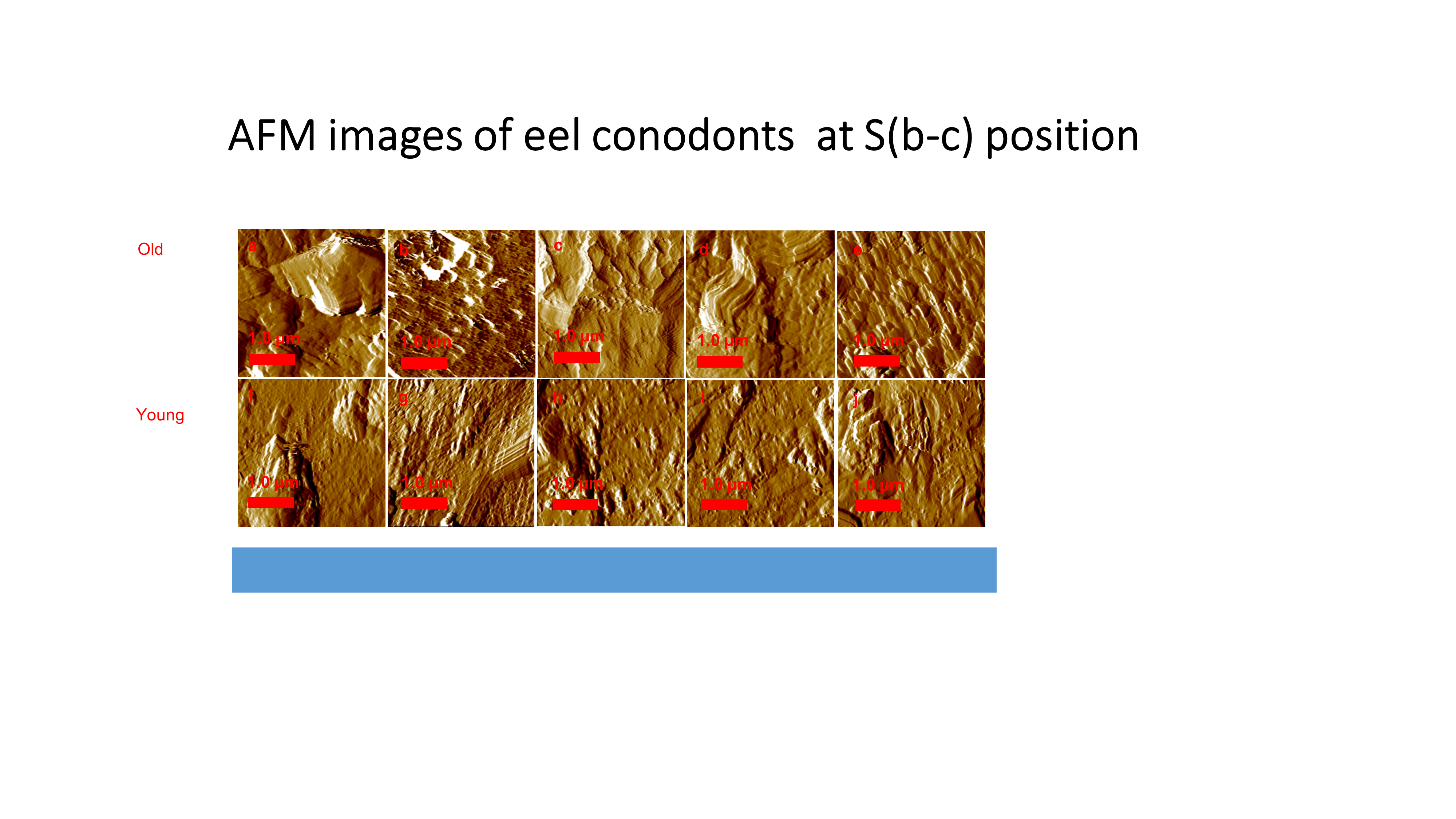


**1**

**2**

**3**

**4**

**5**

**O**

**J**

**Figure S3.** Representative AFM amplitude images of S_b-c_ conodonts, where top (a-e= Zones 1-5) and bottom (f-j= Zones 1-5) rows are for the juvenile and older conodonts, respectively. Left to right rows sequentially show the tip to base segments.

**
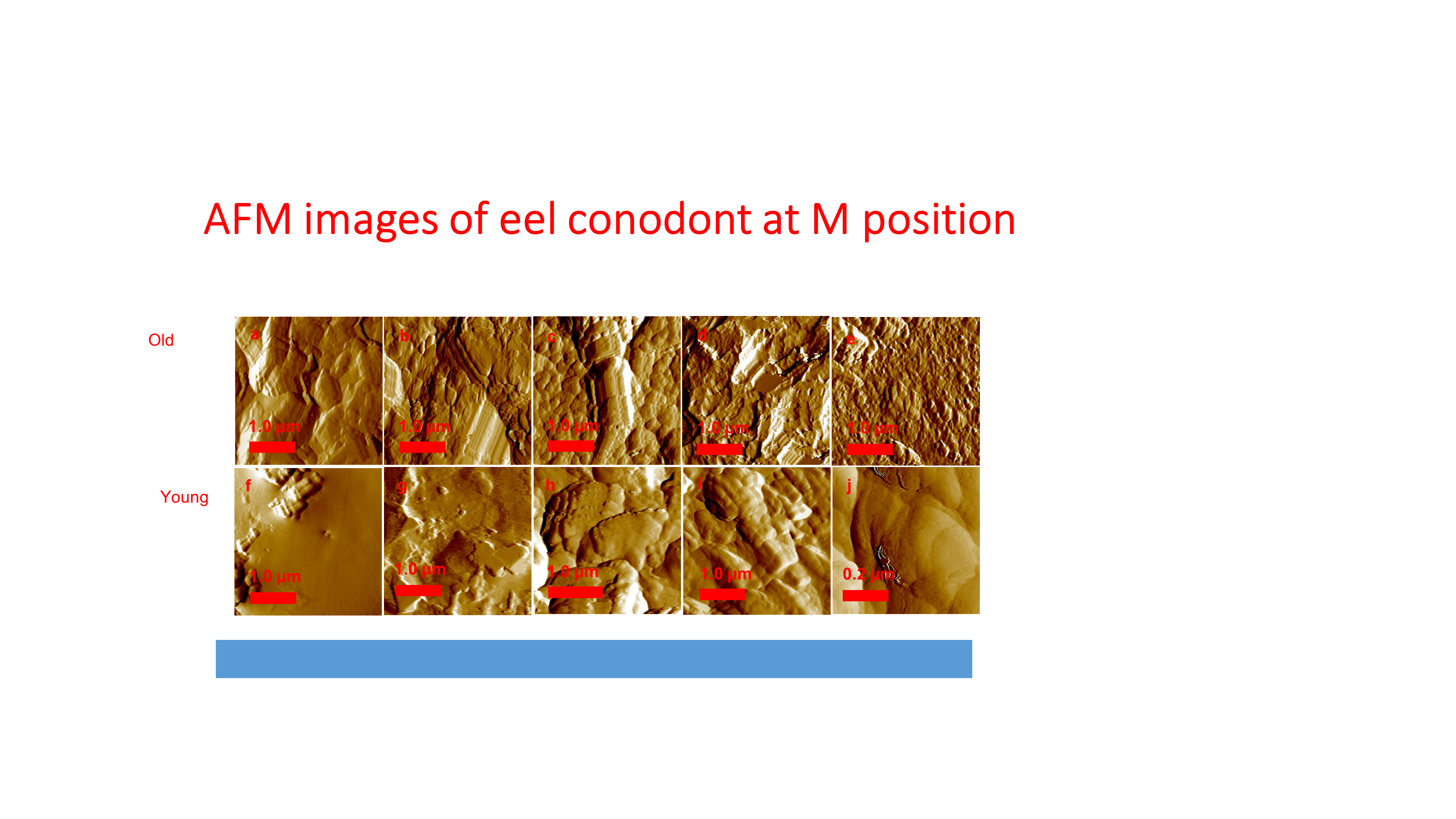
**

**O**

**J**

**5**

**4**

**3**

**2**

**1**

**Figure S4.** Representative AFM amplitude images of M conodonts, where top (a-e: Zones 1-5) and bottom (f-j: Zones 1-5) rows are for the juvenile and older conodonts, respectively. Left to right rows sequentially show the tip to base segments.

**References:**

1. Johnson, K. L.; Kendall, K.; Roberts, A. D., Surface energy and the contact of elastic solids. *Proceedings of the royal society of London. A. mathematical and physical sciences* **1971,** *324* (1558), 301-313.

2. Lee, H. D.; Ray, K. K.; Tivanski, A. V., Solid, Semisolid, and Liquid Phase States of Individual Submicrometer Particles Directly Probed Using Atomic Force Microscopy. *Analytical Chemistry* **2017,** *89* (23), 12720-12726.

3. Hutchins, K. M.; Rupasinghe, T. P.; Oburn, S. M.; Ray, K. K.; Tivanski, A. V.; MacGillivray, L. R., Remarkable decrease in stiffness of aspirin crystals upon reducing crystal size to nanoscale dimensions via sonochemistry. *CrystEngComm* **2019,** *21* (13), 2049-2052.

4. Mohapatra, H.; Kruger, T. M.; Lansakara, T. I.; Tivanski, A. V.; Stevens, L. L., Core and surface microgel mechanics are differentially sensitive to alternative crosslinking concentrations. *Soft matter* **2017,** *13* (34), 5684-5695.
